# Replication of CMV in the gut of HIV-infected individuals and epithelial barrier dysfunction

**DOI:** 10.1371/journal.ppat.1006202

**Published:** 2017-02-27

**Authors:** Ekaterina Maidji, Ma Somsouk, Jose M. Rivera, Peter W. Hunt, Cheryl A. Stoddart

**Affiliations:** 1 Division of Experimental Medicine, Department of Medicine, Zuckerberg San Francisco General, University of California, San Francisco, San Francisco, California, United States of America; 2 Division of Gastroenterology, Department of Medicine, Zuckerberg San Francisco General, University of California, San Francisco, San Francisco, California, United States of America; University of North Carolina at Chapel Hill, UNITED STATES

## Abstract

Although invasive cytomegalovirus (CMV) disease is uncommon in the era of antiretroviral therapy (ART), asymptomatic CMV coinfection is nearly ubiquitous in HIV infected individuals. While microbial translocation and gut epithelial barrier dysfunction may promote persistent immune activation in treated HIV infection, potentially contributing to morbidity and mortality, it has been unclear whether CMV replication in individuals with no symptoms of CMV disease might play a role in this process. We hypothesized that persistent CMV replication in the intestinal epithelium of HIV/CMV-coinfected individuals impairs gut epithelial barrier function. Using a combination of state-of-the-art *in situ* hybridization technology (RNAscope) and immunohistochemistry, we detected CMV DNA and proteins and evidence of intestinal damage in rectosigmoid samples from CMV-positive individuals with both untreated and ART-suppressed HIV infection. Two different model systems, primary human intestinal cells differentiated *in vitro* to form polarized monolayers and a humanized mouse model of human gut, together demonstrated that intestinal epithelial cells are fully permissive to CMV replication. Independent of HIV, CMV disrupted tight junctions of polarized intestinal cells, significantly reducing transepithelial electrical resistance, a measure of monolayer integrity, and enhancing transepithelial permeability. The effect of CMV infection on the intestinal epithelium is mediated, at least in part, by the CMV-induced proinflammatory cytokine IL-6. Furthermore, letermovir, a novel anti-CMV drug, dampened the effects of CMV on the epithelium. Together, our data strongly suggest that CMV can disrupt epithelial junctions, leading to bacterial translocation and chronic inflammation in the gut and that CMV could serve as a target for therapeutic intervention to prevent or treat gut epithelial barrier dysfunction during HIV infection.

## Author summary

Intestinal epithelial barrier dysfunction is a well-known consequence of HIV infection that persists in spite of ART. The underlying mechanisms by which HIV perturbs intestinal epithelial junctions remain unclear, and the impact of opportunistic viral pathogens in the gut has not been fully appreciated. HIV-infected individuals are almost universally coinfected with CMV. While ART has resulted in a dramatic decline in the occurrence of end-organ CMV diseases, CMV remains an independent contributor to systemic inflammation in HIV-infected people. In our analysis of rectosigmoid biopsies from CMV/HIV-coinfected individuals, we found active CMV replication associated with intestinal damage in the gut of ART-suppressed HIV-infected individuals with no symptoms of CMV disease. We demonstrated that CMV productively infects intestinal epithelial cells and, independent of HIV, disrupts their tight junctions and compromises epithelial barrier function. Furthermore, the CMV-induced proinflammatory cytokine IL-6 is a key factor in this process, and attenuation of CMV replication by letermovir, a new anti-CMV agent currently in clinical development, was sufficient to prevent CMV-induced loss of epithelial integrity. Our data highlight the role of CMV as a cofactor in intestinal epithelial barrier dysfunction in asymptomatic HIV infection and suggest a novel treatment strategy to prevent intestinal epithelial barrier dysfunction and inflammation in HIV infection.

## Introduction

Immune activation and intestinal epithelial barrier dysfunction are major hallmarks of HIV infection that persist in spite of potent combination antiretroviral therapy (ART) [1–6]. The barrier properties of mucosal intestinal epithelium are maintained by a monolayer of columnar epithelial cells that are firmly connected by intercellular tight junctions [7]. Disruption of these junctions was proposed as a mechanism for the increased colonic permeability in ART-suppressed patients [8]. In HIV infection, impaired integrity of the intestinal epithelial barrier facilitates bacterial translocation, a major contributor to chronic immune activation [4, 6, 9–11]. The precise mechanisms by which HIV perturbs the intercellular tight junctions of intestinal epithelia remain an active area of investigation [12–14].

Currently, gut epithelial barrier dysfunction during HIV infection is attributed to the increased production of inflammatory cytokines by activated mucosal T cells [15, 16] and by mucosal epithelial cells directly responding to HIV-1 gp120 [17, 18]. However, the cellular sources of inflammatory cytokines that lead to gut barrier dysfunction in HIV-infected individuals remain controversial [16], and the mechanisms described above do not include the presence in the gut of a variety of opportunistic pathogens, specifically cytomegalovirus (CMV), which has repeatedly been suggested as a cofactor for HIV disease progression and persistence of inflammation despite suppressive ART [19–21]. Importantly, the gastrointestinal tract experiences severe CD4^+^ T cell depletion at all stages of HIV disease [22], which is not completely restored in chronically HIV-infected individuals following ART [23–25] and could thus be an important site for local and asymptomatic CMV reactivation.

CMV was a common opportunistic pathogen in HIV-infected individuals before the introduction of ART, accounting for significant morbidity and mortality [26, 27]. While end-organ disease from CMV (e.g., retinitis, colitis, and encephalitis) is rare during ART-mediated viral suppression, CMV appears to contribute to persistent immune activation and inflammation in this setting [28] and is responsible for a sizable proportion of the entire memory T-cell response [29]. CMV often reactivates asymptomatically in ART-suppressed HIV-infected individuals, who are almost universally coinfected with CMV [30], and the virus has a strong physiologic association with aging [29, 31, 32], cardiovascular diseases [20, 33–36], and mortality [37, 38]. The gastrointestinal tract is a major site of CMV disease in people with immune deficiency including individuals with HIV infection [39–43]. CMV targets endothelial, stromal, and intestinal epithelial cells and can cause erosive and ulcerative processes [40, 44–46]. CMV lesions in the gut may compromise mucosal epithelial barrier function, which is maintained by intercellular tight junctions, multiprotein complexes that seal the space between adjacent cells [47]. We and others have previously reported that CMV disrupts the tight junctions of polarized retinal pigment epithelial cells and the adherens junctions of endothelial cells [48–52].

In this study, we used state-of the-art techniques in dual immunohistochemistry (IHC) and *in situ* hybridization (ISH) to show that CMV persistence in the rectosigmoid tissues of asymptomatic CMV-positive individuals with both untreated and ART-suppressed HIV infection was associated with gut epithelial barrier dysfunction. We used two different model systems to investigate the permissiveness of intestinal epithelial cells to CMV replication: primary human intestinal cells differentiated *in vitro* to form polarized monolayers and a mouse model of the human gut. We found that independent of HIV, CMV disrupts tight junctions of polarized intestinal cells, significantly reducing transepithelial electrical resistance (TER), a measure of epithelial monolayer integrity, and enhancing epithelial barrier permeability. Furthermore, CMV-associated disruption of the intestinal epithelium integrity could be attributed, at least in part, to the CMV-induced proinflammatory cytokine interleukin-6 (IL-6). Importantly, letermovir, a novel anti-CMV drug currently in clinical development, preserved epithelial polarity in this system. Our results support a potentially active role for CMV in driving epithelial barrier dysfunction and microbial translocation. Taken together, these observations underscore a novel way to prevent and treat gut epithelial barrier dysfunction in HIV infection.

## Results

### CMV persists in rectosigmoid biopsies from CMV-positive individuals with untreated as well as ART-suppressed HIV infection

We analyzed rectosigmoid biopsies from CMV/HIV-coinfected individuals for CMV replication by IHC and RNAscope ISH. All these individuals had no symptoms or suspicion of CMV disease. To determine whether HIV-status is associated with an increase of CMV detection in the gut, a HIV-negative CMV-positive control group was included in the study. Clinical characteristics of the study cohort and the results of CMV detection are individually described in Table 1.

**Table 1:** Characteristics of clinical samples.

Sample ID	HIV infection status	ART (years)	CD4^+^ T cell count (cells/μl)	CD4^+^ T cell nadir count (cells/μl)	CMV IgG	Gut HIV RNA (ISH)	Gut CMV DNA (ISH)	Gut CMV Proteins (IHC)
1878*	Uninfected	N/A	ND	ND	−	−	−	0
1916*	Uninfected	N/A	ND	ND	−	−	−	0
1939*	Uninfected	N/A	ND	ND	−	−	−	0
1958*	Uninfected	N/A	ND	ND	−	−	−	0
1992*	Uninfected	N/A	ND	ND	−	−	−	0
1298	Untreated	0	208	260	+	+	+	405 ± 45^a^
1323	Untreated	0	679	550	+	+	+	166 ± 33^a^
1747	Untreated	0	53	83	+	+	+	355 ± 11^a^
1036	ART	3	465	220	+	−	−	0
2118	ART	12	93	21	+	+	−	0
2021	ART	13	404	35	+	+	+	1050 ± 11^a^
2031	ART	13	242	20	+	+	−	0
5009	ART	15	239	100	+	−	+	633 ± 66^a^
3022	ART	19	301	90	+	+	−	0
2119	ART	20	265	12	+	+	+	483 ± 68^a^
3040	ART	20	305	90	+	−	−	0
3054	ART	21	293	4	+	+	−	0
3010	ART	21	1110	160	+	−	−	0
2067	ART	22	528	30	+	−	−	0
2013 (9/19/11)^§^	ART	21	777	13	+	+	+	263 ± 65^a^
2013 (3/4/13)^§^	ART	23	552	13	+	+	+	943 ± 91^a^
3068	ART	23	515	149	+	+	+	230 ± 37^a^
2026	ART	24	578	120	+	−	+	473 ± 32^a^
3025	ART	24	205	39	+	−	−	0
2180	ART	25	249	9	+	−	+	350 ± 57^a^
3091	ART	25	121	32	+	+	+	280 ± 34^a^
4001	ART	31	538	197	+	−	−	0
3037	ART	32	451	400	+	+	+	500 ± 52^a^
1807	Uninfected	N/A	N/A	N/A	+	ND	ND	+^b^
1930 (06/15/15)^§^	Uninfected	N/A	N/A	N/A	+	ND	ND	+^b^
1930 (07/27/15)^§^	Uninfected	N/A	N/A	N/A	+	ND	ND	+^b^
6532	Uninfected	N/A	N/A	N/A	+	ND	ND	+^b^
S16-3170A^#^	Uninfected	N/A	N/A	N/A	+	ND	ND	+^c^
S16-3170B^#^	Uninfected	N/A	N/A	N/A	+	ND	ND	−

ART, antiretroviral therapy; ISH, *in situ* hybridization; IHC, immunohistochemistry; ND, not determined because of limitations in the number of available tissue sections; N/A, not applicable

* healthy individuals

^§^ gut biopsies obtained from the same individual

^#^ multiple gut biopsies from an individual immunosuppressed for lupus with CMV enteritis and colitis before (A) and after (B) treatment with valganciclovir

^a^ foci/mm^2^ (mean ± SE, n = 10)

^b^ CMV proteins were detected in cells neighboring the intestinal crypts but not within intestinal epithelial cells

^c^ numerous CMV immediate early (IE) protein-positive cytomegalic cells.

**Fig 1:**
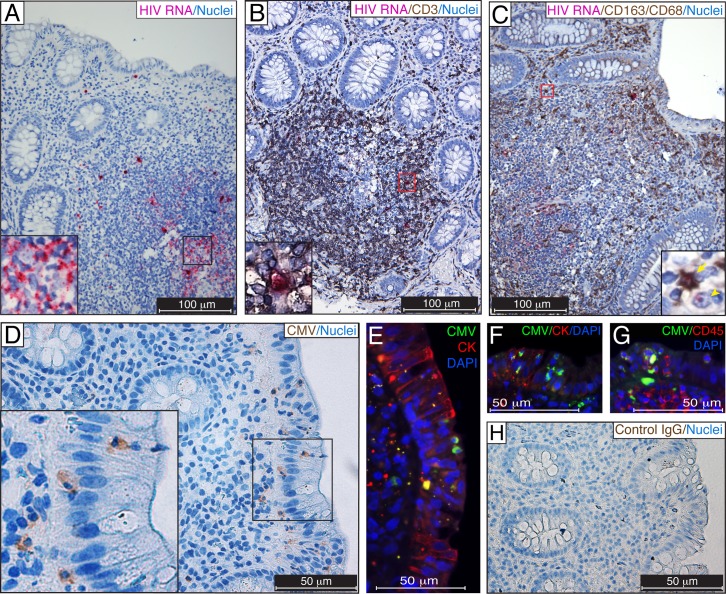
Detection of CMV in rectosigmoid biopsies from untreated HIV/CMV-coinfected individuals. (**A**–**C**) Formalin-fixed paraffin-embedded (FFPE) rectosigmoid biopsies were probed for HIV RNA (fuchsia) by RNAscope ISH. CMV proteins were detected in adjacent tissue sections by chromogenic (**D,** brown) and fluorescent (**E**−**G**, green) IHC using a blend of anti-CMV monoclonal antibodies (Millipore) comprising clone 8B1.2 reacting with an IE protein of 68–72 kDa, clone 1G5.2 reacting with unspecified late CMV protein, and clone 2D4.2 reacting with a late structural protein of 47–55 kDa. The adjacent section of **D** was immunostained with isotype control IgG (**H**). Regions of interest outlined by black (**A**, **D**) or red (**B**, **C**) are shown in the insets. HIV-infected T cells were identified using CD3 detection (**B,** dark brown), and HIV-infected macrophages were identified using CD163 and CD68 markers (**C,** dark brown). Putative leukocytes containing CMV structural proteins (**D**, brown and **E**, **F**, green) appeared to be trafficking across the intestinal epithelium identified by cytokeratin (CK, red) immunostaining. Leukocyte phenotype was verified by coimmunostaining for CMV proteins (**G**, green) and CD45 (**G**, red). Nuclei were counterstained with hematoxylin (**A**−**D**, **H**) or DAPI (**E**−**G**). A HIV-infected CD163^+^CD68^+^ macrophage (arrow) and CD163^−^CD68^−^ putative T cell (arrowhead) are shown in panel **C**. Scale bars: 100 μm (**A**–**C**), 50 μm (**D**–**H**). These results are representative of those observed in rectosigmoid biopsies from 3 untreated HIV-infected individuals with asymptomatic CMV infection.

First, we identified regions of HIV-1 replication in biopsy samples from untreated CMV/HIV-coinfected individuals by RNAscope. Thymic organoid tissue from a HIV-infected humanized BLT mouse was used as a positive control for HIV detection [53] (S1 Fig). HIV RNA was detected in numerous cells residing in gut-associated lymphoid tissue (GALT) (Fig 1A), and a majority of these cells were CD3-positive T cells (Fig 1B). Numerous CD163^+^CD68^+^ macrophages, often containing HIV RNA, were detected near intestinal crypts (Fig 1C), and some of these macrophages revealed a single HIV transcript (red dot) in the nucleus (Fig 1C, inset), indicating potential productive HIV replication. HIV RNA-positive cells were detected in rectosigmoid biopsies from all 3 asymptomatic CMV-positive individuals with untreated HIV infection that were examined, but not in biopsy samples from 5 HIV-negative CMV-negative individuals (S1F and S1G Fig).

**Fig 2:**
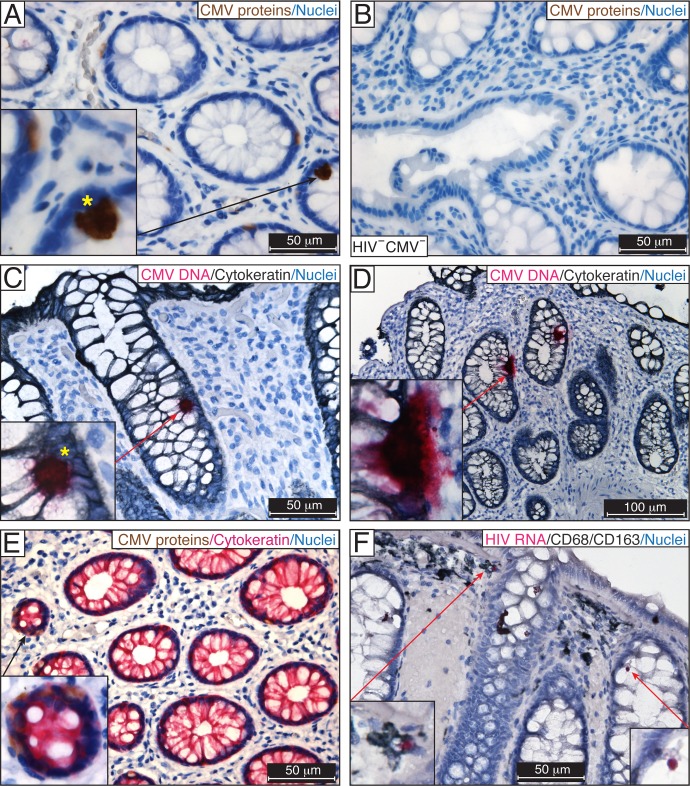
Detection of CMV in rectosigmoid biopsies from ART-suppressed HIV/CMV-coinfected individuals. IHC for CMV IE, early and late structural proteins using the anti-CMV antibody blend in FFPE rectosigmoid biopsies from ART-suppressed HIV/CMV-coinfected individuals (**A**) and a HIV-negative, CMV-negative individual **(B**). The presence of CMV was verified by DNAscope ISH using a probe targeting the noncoding region (120742–122152) of CMV strain Merlin, which corresponds to the UL83 (pp65) sequence (fuchsia) (**C, D**). Yellow stars indicate similar patterns of IE protein expression (**A**) and the presence of viral DNA (**C**) in the nuclei of CMV-infected intestinal cells. (**E**) CMV early and late proteins (brown) were detected in the cytoplasm of intestinal epithelial cells costained for cytokeratin (fuchsia). (**F**) HIV RNA was randomly detected by RNAscope ISH in CD68/CD163-positive macrophages (insets) in an adjacent section of tissue shown in (**C**). Nuclei were counterstained with hematoxylin. Scale bars: 50 μm (**A**–**C**, **E**, **F**), 100 μm (**D**). These results are representative of those observed in rectosigmoid biopsies from 9 asymptomatic ART-suppressed HIV/CMV-coinfected individuals.

Next, we assessed whether CMV infection coexisted in rectosigmoid biopsies from individuals with untreated HIV infection. CMV structural proteins were detected in the cytoplasm of intestinal epithelial cells and in leukocytes within the intestinal epithelium (Fig 1D–1G). Immunostaining for CMV proteins was verified by both chromogenic (Fig 1D) and fluorescent (Fig 1E–1G) IHC. The specificity of detection of CMV proteins was verified by the lack of immunostaining with an isotype-control IgG (Fig 1D & 1H, matching regions of interest of adjacent sections). CMV was detected in rectosigmoid biopsies from all 3 CMV-positive individuals with untreated HIV infection that were examined (Table 1), and CMV proteins were not detected in biopsy samples from 5 HIV-negative, CMV-negative individuals (Fig 2B).

We were surprised to observe clear evidence of CMV infection in the rectosigmoid biopsies from ART-suppressed HIV/CMV-coinfected individuals, although CMV was not present in all samples. CMV IE proteins, which are essential for CMV replication, were evident in the nuclei of numerous intestinal epithelial cells in 9 out of 19 samples (Fig 2A). Intestinal epithelial cells that were identified by cytokeratin costaining frequently contained CMV proteins in the cytoplasm (Fig 2E, inset). The specificity of CMV detection was verified by the absence of CMV staining in gut biopsies from HIV-negative CMV-negative individuals (Fig 2B) and by the lack of immunostaining of the adjacent section with an isotype control IgG (Fig 1D & 1H, matching regions of interest of adjacent sections). We further assessed the extent of CMV infection in rectosigmoid biopsies from ART-suppressed individuals by DNAscope ISH (Fig 2C). A human fetal lung explant inoculated ex vivo with CMV was used as a positive control for CMV detection [54] (S2 Fig). Abundant CMV DNA was detected in numerous intestinal epithelial cells outlined by cytokeratin immunostaining (Fig 2C and 2D). Importantly, the pattern of CMV DNA often had remarkable similarities to those we observed for CMV IE expression detected by IHC (Fig 2C and 2A, yellow stars). Similar results were observed using probes targeting CMV IE (S2A Fig) and CMV pp65 coding sequences (S2B Fig). Using two methods, IHC and DNAscope ISH, CMV was detected in biopsies from 9 of 19 (47%) asymptomatic ART-suppressed HIV/CMV-coinfected individuals (Table 1). We also examined the level of HIV replication in the rectosigmoid biopsies from ART-suppressed HIV/CMV-coinfected individuals by RNAscope ISH. Single HIV RNA-positive cells were occasionally detected in these biopsies (Fig 2F) in contrast to rectosigmoid biopsies from CMV-positive individuals with untreated HIV infection (Fig 1A–1C). CD68/CD163-positive macrophages containing HIV RNA (Fig 2F, insets) were occasionally detected in regions adjacent to those containing CMV DNA (Fig 2C). Among ART-suppressed individuals, 64% had evidence of HIV replication in samples with detectable CMV activity, whereas 33% had evidence of HIV replication in samples without detectable CMV, although this difference was not statistically significant (P = 0.18).

CMV infection in the intestine of individuals with inflammatory bowel diseases has been frequently correlated with an increased production of IL-6 [55], and CMV shedding was associated with higher IL-6 levels in vaginal swabs of ART-suppressed HIV-infected women [56]. Therefore, we measured IL-6 in plasma samples obtained from the study participants immediately prior to biopsy. A trend toward higher IL-6 levels was found in the plasma of those ART-suppressed HIV/CMV-coinfected individuals whose rectosigmoid biopsies had evidence of CMV activity compared to gut samples from those without evidence of CMV activity (median 2.6 versus 1.0 pg/ml, Mann Whitney, one-tailed P = 0.16) or those who were HIV- and CMV-seronegative (median 2.6 versus 1.4 pg/ml, Mann Whitney, one-tailed P = 0.15), although the differences were not statistically significant.

**Fig 3:**
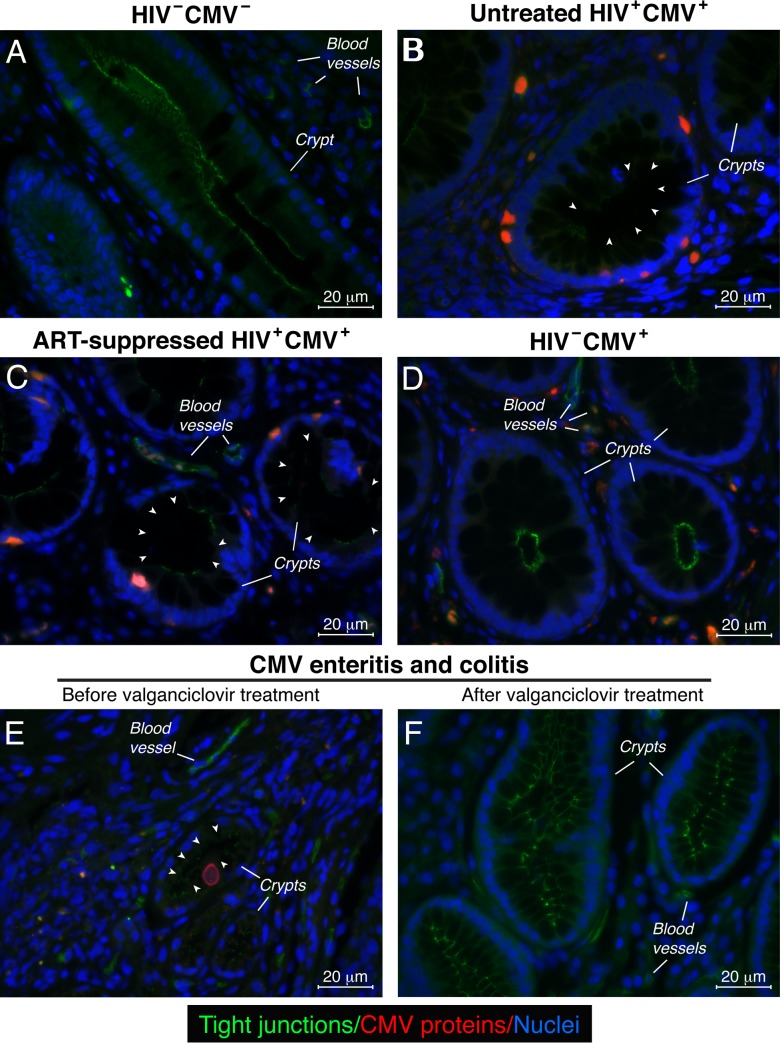
Disruption of intestinal tight junctions was associated with the presence of CMV in rectosigmoid biopsies. Immunolocalization of ZO-1, a marker of tight junctions (tight junctions, green) and CMV IE, early, and late proteins (red) in FFPE rectosigmoid biopsies by fluorescent IHC. ZO-1 labeling appeared in the epithelial cells of the intestinal crypts and endothelial cells of blood vessels. Results are representative of those observed in rectosigmoid biopsies from 5 HIV-negative CMV-negative (HIV^−^CMV^−^) individuals (**A**), 3 untreated HIV/CMV-coinfected (HIV^+^CMV^+^) individuals (**B**), 9 ART-suppressed HIV/CMV-coinfected (HIV^+^CMV^+^) individuals (**C**), 3 HIV-negative CMV-positive (HIV^−^CMV^+^) individuals (**D**) and an individual with active CMV infection before (**E**) and after (**F**) treatment with valganciclovir. The arrowheads show areas of discontinuous tight junctions (absence of ZO-1 staining). Nuclei were counterstained with DAPI. Scale bars: 20 μm

IL-6 is a pleiotropic cytokine that can undermine the integrity of the intestinal barrier [57]. We next assessed whether CMV infection in the gut coexists with disrupted epithelial barriers. Tissue sections of the rectosigmoid biopsies described in Table 1 were coimmunostained for CMV proteins and zonula occludens-1 (ZO-1), a marker of tight junctions (Fig 3). ZO-1 was confined to the luminal apical surface of the intestinal epithelium and appeared uniformly distributed in the rectosigmoid biopsies from HIV-negative CMV-negative individuals (Fig 3A). In contrast, in rectosigmoid biopsies from 3 untreated HIV/CMV-coinfected individuals, ZO-1 appeared discontinuous and vague in the crypt containing CMV-infected epithelial cells (Fig 3B). In 9 rectosigmoid biopsies from ART-suppressed HIV/CMV-coinfected individuals, CMV detection in intestinal epithelial cells was accompanied by disruption of the continuity of ZO-1 (Fig 3C). Interestingly, ZO-1 staining was intact and continuous in the crypts of rectosigmoid biopsies from 4 HIV-negative CMV-positive individuals (Fig 3D). Although CMV proteins were detected in those biopsies in cells surrounding intestinal crypts, no CMV proteins were detected in the intestinal epithelial cells (Table 1), and epithelial integrity was not disturbed. To confirm our finding that disruption of intestinal tight junctions is specific to CMV, we examined multiple gut biopsies from an individual with clinically diagnosed CMV enteritis and colitis before and after valganciclovir treatment. Numerous CMV IE-positive cytomegalic cells were detected in the intestinal crypts in all 4 pretreatment biopsies (Table 1). Notably, those cytomegalic cells were often lifted from the level of the intestinal monolayer into the lumen, exhibiting damage of the epithelial barrier (Fig 3E). The staining for ZO-1 in those crypts had decreased intensity in the tight junction regions and often was discontinuous. ZO-1 localization was diffuse or lost in the detaching cytomegalic cells and had remarkable similarity to the CMV-infected crypts observed in the rectosigmoid biopsies from untreated and ART-suppressed HIV/CMV-coinfected individuals (Fig 3B and 3C). Importantly, after valganciclovir treatment, ZO-1 immunolabeling was intact, continuous, and strictly limited to the tight junction regions of the intestinal crypts, and no CMV was detected (Fig 3F).

Taken together, these data indicate that CMV persists in the intestinal epithelium of CMV-positive individuals with both untreated and ART-suppressed HIV infection and is associated with the disruption of epithelial tight junctions.

### CMV productively replicates in the intestinal epithelium

**Fig 4:**
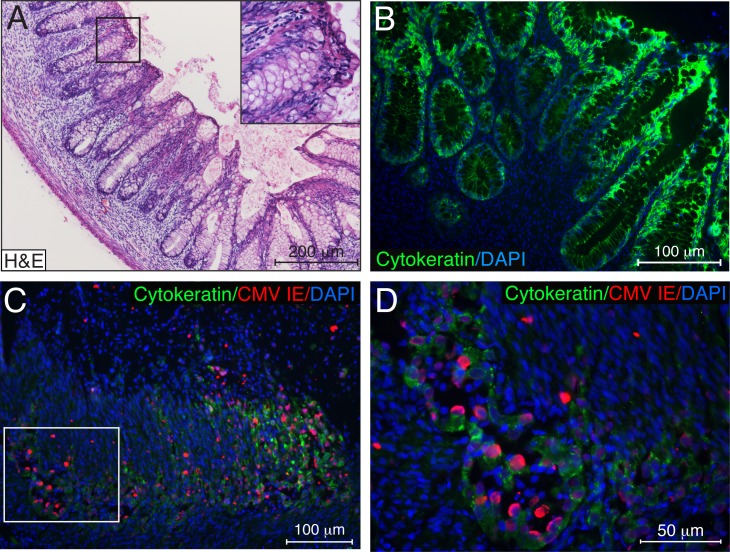
CMV infection rapidly depletes epithelial cells of the human fetal intestine differentiated *in vivo*. (**A**, **B**) Human fetal intestine implanted subcutaneously into SCID mice differentiates *in vivo* over 4 weeks. Gut implant cross-sections stained with hematoxylin and eosin (H&E) (**A**) and immunostained for human cytokeratin (**B**, green). (**C**) Histologic sections of gut implants 7 days after CMV VR1814 inoculation with 5.7 log_10_ IU were coimmunostained for CMV IE proteins (red) and human cytokeratin (green). The region of interest outlined by the white oblong is magnified in (**D**). Data are representative of those obtained in 3 independent experiments using gut tissues from three donors. Nuclei were counterstained with hematoxylin (**A**) and DAPI (**B**−**D**). Scale bars: 200 μm (**A**), 100 μm (**B**, **C**), 50 μm (**D**).

To further investigate CMV pathogenesis in the intestinal epithelium, we developed a SCID-hu gut mouse model. Human fetal gut subcutaneously implanted into severe-combined immunodeficient (C.B17 scid) (SCID) mice developed a lumen with a morphologically precise mucosal layer containing villi and crypts, lamina propria, and muscularis layers during 4 weeks *in vivo* (Fig 4A). The mucosal epithelium of the villi and crypts was composed of a single layer of intestinal epithelial cells expressing human cytokeratin (Fig 4B). We analyzed 34 intestinal xenografts originating from 4 fetal donors and in all cases, the fetal intestine had matured into differentiated human intestine by 4 weeks after implantation. At this stage of development, gut implants were intraluminally inoculated with 5.7–6.4 log_10_ infectious units (IU) of CMV VR1814. Using immunofluorescence techniques, we observed substantial CMV infection with marked mucosal damage (Fig 4C) 7 days after inoculation. During this period, CMV spread throughout the entire intestinal epithelial layer, depleting cytokeratin-positive epithelial cells (Fig 4C). Cytomegalic IE-positive epithelial cells appeared to be detaching into the lumen of the crypts (Fig 4D), exhibiting notable similarity to the detaching cytomegalic cells detected in the gut of an individual with active CMV infection (Fig 3E). These data indicate that the intestinal epithelium is highly susceptible to CMV infection.

**Fig 5:**
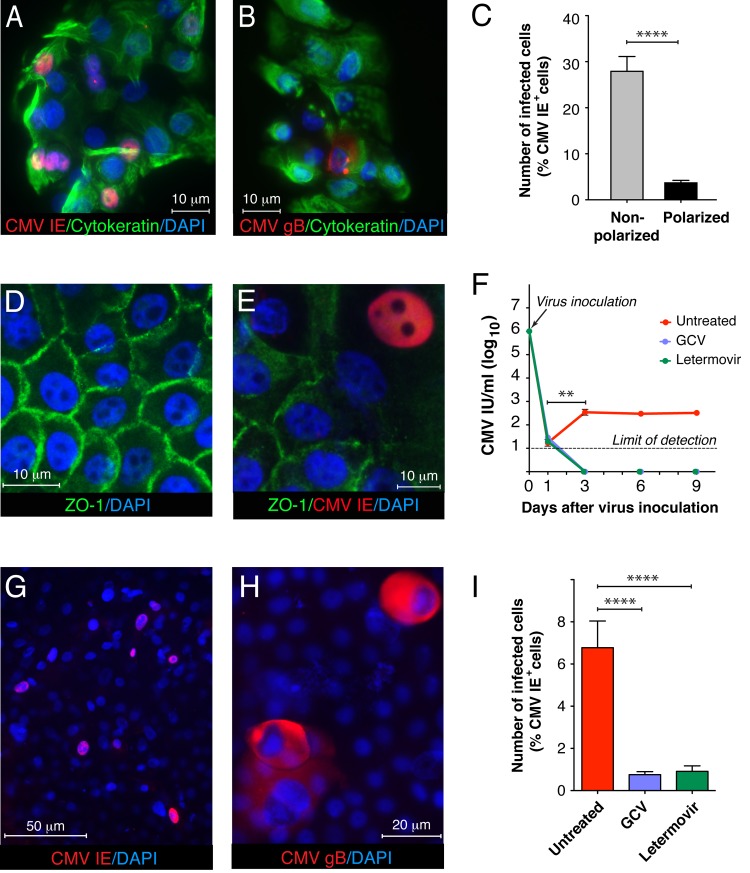
CMV productively infects human intestinal epithelial cells. Nonpolarized HCoEpiC infected with CMV VR1814 at a MOI of 1.0 expressed cytokeratin, an epithelial cell marker (green) and CMV IE (**A,** red) at 1 day and gB (**B,** red) at 3 days after inoculation. When plated on collagen-coated transwells for 6 days, HCoEpiC differentiated into a polarized monolayer expressing ZO-1 (**D**, green). (**C**) Nonpolarized (**A**) and polarized HCoEpiC (**E**) were inoculated from the apical surface with CMV VR1814 at a MOI of 1.0. At day 1 after inoculation, cells were fixed and immunostained for CMV IE, and nuclei were counterstained with DAPI. Images of the infected cells were acquired at magnification 400x, and a percentage of CMV IE-positive nuclei were calculated relative to the total number of DAPI-stained nuclei per image. (**F, I**) Polarized HCoEpiC were inoculated with CMV VR1814 at a MOI of 1.0. After 1 h virus adsorption, some infected cell cultures were treated with an inhibitory concentration (50 nM) of letermovir, whereas other cultures were treated with an inhibitory concentration (20 μM) of GCV. Infectious viral progeny in the medium at 1, 3, 6 and 9 days as well as in the inoculum (day 0) were quantified in human fibroblasts as the indicator monolayer (**F**). (**G**, **I**) CMV slowly replicated in polarized HCoEpiC during 9 days as indicated by the low number of CMV IE-positive cells (6.7%), which was nevertheless higher than CMV-IE-positive cells treated with GCV or letermovir (****P<0.0001). (**H**) Cytomegalic cells expressing CMV gB (red) were detected on top of the polarized HCoEpiC monolayer 9 days after inoculation. Nuclei were counterstained with DAPI. Scale bars: 10 μm (**A, B, D, E**), 50 μm (**G**), 20 μm (**H**). Data are shown as the mean ± SEM with n = 10 (**C, I**) and n = 4 (**F**). Statistical comparison was performed by the Mann Whitney U-test and was considered significant at ****P<0.0001 (**C, I**); **P<0.01 (**F**, CMV infectious units (IU)/ml at day 3 versus day 1).

To recapitulate CMV infection in rectosigmoid tissues, we devised an *in vitro* polarized cell model with primary human colon epithelial cells (HCoEpiC). These cells retain the morphological and functional properties of intestinal epithelial cells during the first four passages. They expressed cytokeratin, an epithelial cell marker (S3A Fig), and were negative for vimentin, a mesenchymal cell marker (S3C Fig). When HCoEpiC were inoculated with CMV at a multiplicity of infection (MOI) of 1.0, approximately 30% of the cells expressed CMV IE at day 1 (Fig 5A and 5C) and at day 3, some infected cells expressed the CMV envelope glycoprotein gB, indicating productive infection (Fig 5B). The epithelial origin of the infected cells was verified at each passage by coimmunostaining for cytokeratin (S3B Fig, Fig 5A and 5B).

Once HCoEpiC cells were plated on collagen-coated transwells for 6 days, they differentiated forming polarized monolayers and expressed ZO-1 in a ring-like pattern (Fig 5D). When polarized HCoEpiC were inoculated with CMV at a MOI of 1.0, only an average of 3.7% of the cells expressed CMV IE at day 1 (Fig 5C and 5E). Thus, the susceptibility of polarized HCoEpiC to CMV infection was significantly lower as compared to nonpolarized cells (P<0.0001). Furthermore, we found that polarized HCoEpiC sustained a persistently low level of CMV replication as indicated by infectious virus released into the culture medium at approximately 2.5 log_10_ IU per ml during 3–9 days after virus inoculation (Fig 5F). Although this titer of infectious virus is very low relative to the high level of virus in the inoculum (day 0), it was significantly higher than residual input virus detected at day 1. Moreover, no infectious virus was detected when virus replication was suppressed with the reference compound ganciclovir (GCV), a widely used polymerase inhibitor for the treatment of CMV infection and the novel small-molecule CMV viral terminase inhibitor AIC246 (letermovir) [58]. The intriguing observation of persistent low-level CMV replication in polarized HCoEpiC could result from the distinctive feature of intestinal epithelial cells to detach into the crypt lumen with the loss of polarity, thus exposing additional target cells to CMV infection (Fig 3E, Fig 4D). Indeed, 9 days after inoculation, we detected only about 7% of IE-positive cells (Fig 5G and 5I), but cytomegalic cells abundantly expressing CMV gB were detected above the polarized monolayer showing their detaching capacity. Despite the low number of CMV IE-positive cells at day 9 after inoculation, it was significantly higher than in the presence of GCV or letermovir (Fig 5I). These data indicate that although CMV preferentially infects nonpolarized intestinal cells, it is capable of maintaining a low level of replication in highly differentiated polarized intestinal cells.

Taken together, these *in vivo* and *in vitro* experiments showed that human intestinal epithelial cells are fully permissive for CMV replication.

### CMV infection of polarized human colon epithelial cells impairs tight junction integrity, reducing TER and enhancing epithelial barrier permeability

**Fig 6:**
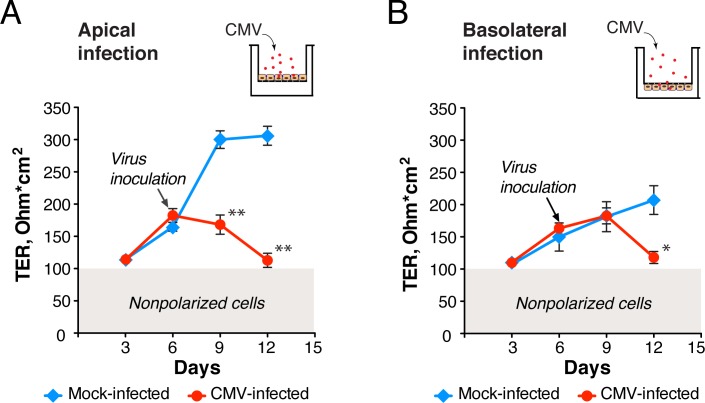
CMV infection decreases TER of polarized HCoEpiC. At 6 days after plating, polarized HCoEpiC were inoculated with CMV VR1814 from the apical surface at a MOI of 1.0 (**A**), from the basolateral surface at a MOI of 10 (**B**), or mock-infected. TER was then measured by Millicell-ERS every 3 days. The shaded area (gray) indicates TER of nonpolarized cells (<100 Ohm*cm^2^ as confirmed by FITC-dextran leakage). Data are shown as the mean ± SEM with n = 5 for each experimental condition. Statistical comparison was performed by the Mann Whitney U-test and was considered significant at (**A**) **P<0.01 and (**B**) *P<0.05.

The association of CMV with the disruption of intercellular junctions of retinal pigment epithelial cells [48–50] and endothelial cells [51, 52] prompted us to examine the effect of CMV infection on the integrity of the intestinal epithelium. When HCoEpiC were grown on collagen-coated porous membranes of transwells that allow access to the culture medium from both the apical and basolateral plasma membranes, they differentiated into a highly polarized epithelial monolayer. Cells formed a sealed epithelium with closed tight junctions (Fig 5D) and developed a considerable TER (>100 Ohm*cm^2^) over 6 days (Fig 6A), mirroring intestinal epithelium in humans. Although TER of the HCoEpiC monolayers continued to increase, we observed the formation of multilayered crypt-like structures and invaginations after 12 days, which hampered further study of epithelial permeability. We therefore performed all experiments within the first 12 days when the cells formed a single polarized monolayer.

To examine the role of polarity in the infection process, we applied the virus inoculum either to the apical surface of polarized cells or to their basolateral surface. For this reason, HCoEpiC were grown on the upper or lower surface of the transwell membrane to orient upward the apical or basolateral side of the monolayer, respectively (Fig 6). For apical infection (Fig 6A), TER of mock-infected HCoEpiC cells continuously increased after formation of a dense monolayer with well-established tight junctions 6 days after seeding (Fig 5D). In contrast, TER of CMV-infected cells declined almost 3-fold (P<0.01) over the 6 days after virus inoculation (Fig 6A) and was accompanied by the disappearance of a ring-like pattern of ZO-1 (Fig 5E). For the study of basolateral infection, we used a 10-fold higher virus inoculum than applied to the apical surface and measured the infectivity of virus passed over blank collagen-coated transwell membranes to ensure that comparable numbers of virions reached the basolateral surface of the HCoEpiC monolayers, as we reported earlier [51]. Infection of HCoEpiC from the basolateral side also significantly reduced TER by almost half (P<0.05) over 6 days of infection (Fig 6B). Thus, we found that CMV VR1814 infected polarized HCoEpiC from both sides, significantly decreasing their TER. However, HCoEpiC developed monolayers with higher TER when they were seeded on the upper surface for apical infection (Fig 6A), so apical infection was chosen for all following experiments.

**Fig 7:**
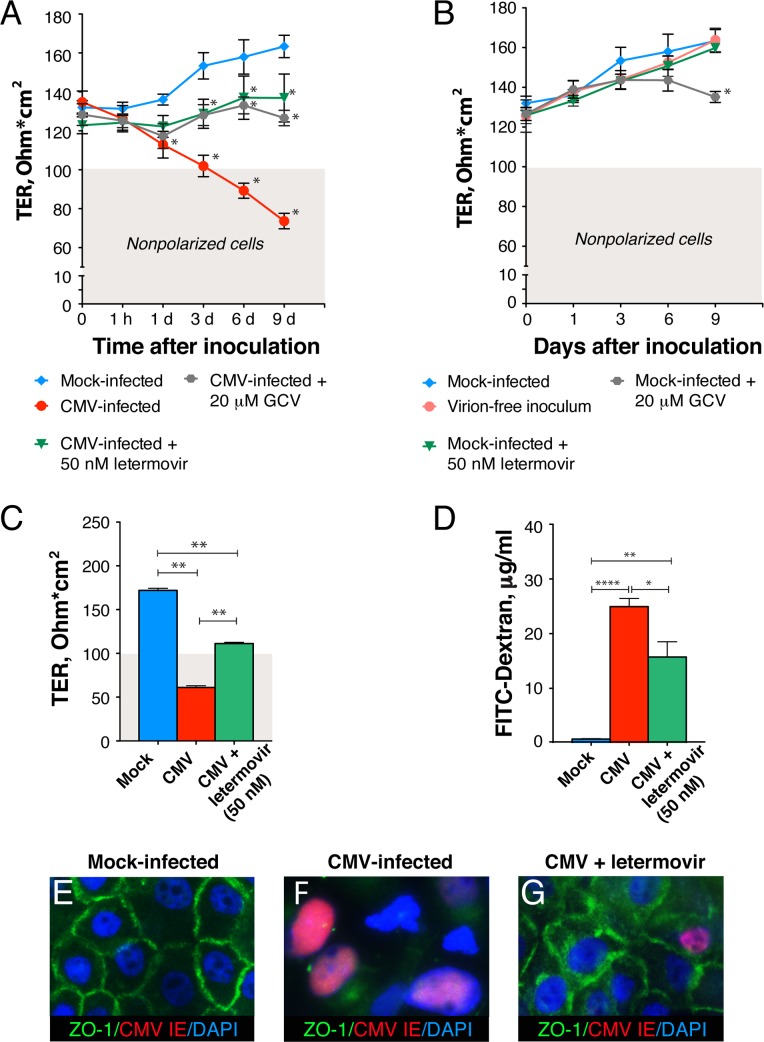
CMV infection decreases integrity of polarized HCoEpiC and increases barrier permeability. (**A, B**) Polarized HCoEpiC were inoculated with CMV VR1814 at a MOI of 1.0 or mock infected. After 1 h virus adsorption, some infected (**A**) and mock-infected (**B**) cell cultures were treated with an inhibitory concentration (50 nM) of letermovir, whereas others were treated with an inhibitory concentration (20 μM) of GCV. Some mock-infected cell cultures were infected with virion-free inoculum filtered through Millex syringe filter units with 0.1 μm pore size (**B**). (**C**, **D**) Polarized HCoEpiC were inoculated with CMV VR1814 at a MOI of 5.0 or mock infected. After inoculation, some infected cells were treated with an inhibitory concentration of letermovir (50 nM). At day 9 after inoculation, TER was measured (**C**), and FITC-dextran was added to the apical chamber. FITC-dextran concentrations were measured 2 h later in the medium from the basolateral chamber (**D**). Shaded areas in (**A**−**C**, gray) indicate TER of nonpolarized cells. Data are shown as the mean ± SEM with n = 4 (**A**, **B**), n = 6 (**C**), and n = 3 (**D**). Statistical comparison was performed by the Mann Whitney U-test and was considered significant at (**A**) *P<0.05 (CMV infected versus mock-infected at day 1, 3, 6 and 9; CMV infected versus CMV infected and treated with letermovir at day 3, 6 and 9; CMV infected versus CMV infected and treated with GCV at day 6 and 9); (**B**) *P<0.05 (mock-infected versus mock-infected treated with GCV at day 9); (**C**) **P<0.01; (**D**) *P<0.05, **P<0.01, ****P<0.0001. IHC for ZO-1 and CMV IE proteins in polarized HCoEpiC that were mock-infected (**E**), infected with CMV and untreated (**F**), and infected and treated with 50 nM letermovir (**G**) for 9 days. Nuclei were counterstained with DAPI, original magnification, x630.

To determine whether virus replication is required for the disruption of intestinal epithelial tight junctions, we inhibited CMV replication with GCV and letermovir (Fig 7A). Polarized HCoEpiC were mock or CMV infected at a MOI of 1.0, and TER was monitored during the course of infection. The TER of CMV-infected untreated cells decreased gradually over the course of infection starting from 1 h after virus inoculation, although differences relative to the mock-infected control reached statistical significance only at day 1 (P<0.05). Treatment of polarized HCoEpiC after CMV inoculation with an inhibitory concentration of GCV and letermovir remarkably prevented significant declines in monolayer resistance over the 9-day period (P<0.05). The effect of both drugs was sufficient to maintain epithelial cell polarity at TER >100 Ohm*cm^2^. Although both drugs had no effect on the TER of mock-infected cells during the first 3 days after treatment, the TER of mock-infected cells treated with GCV significantly declined at day 9 (P<0.05) but not of cells treated with letermovir (Fig 7B). This effect of GCV on the TER of mock-infected cells was reflected by the slight decline in TER at day 9 in infected cells that were otherwise protected by GCV treatment (Fig 7A). No changes in monolayer resistance were observed when cells were infected with virus inoculum filtered through Millex syringe filter units with 0.1 μm pore size to remove CMV virions. These results indicated that the virus inoculum contains no factors that could affect monolayer integrity and that CMV itself induced a decline in TER (Fig 7B).

We further examined the effect of CMV on epithelial permeability by monitoring the ability of a fluorescently labeled, inert dye (FITC-dextran) to migrate from the apical transwell compartment to the lower compartment. Consistent with the results of the TER studies (Fig 7C), a significant increase in FITC-dextran concentration in the lower chamber (P<0.0001) was observed in CMV-infected cells compared with mock-infected cells (Fig 7D). Treatment with letermovir moderately prevented CMV-induced FITC-dextran leakage across polarized HCoEpiC monolayers (P<0.05) but did not block it to the level of mock-infected controls. Mock-infected cells displayed well-defined epithelial junctions 9 days after inoculation as indicated by ZO-1 expression in a ring-like pattern (Fig 7E). CMV-infected polarized HCoEpiC lacked ZO-1 expression indicating the entire disassembly of epithelial junctions (Fig 7F). Notably, CMV-infected polarized HCoEpiC treated with letermovir occasionally expressed CMV IE but retained tight junctions, although a fraction of ZO-1 was redistributed in the cytoplasm (Fig 7G).

Altogether, these results showed that CMV infection disrupted the tight junctions of polarized colon epithelial cells, reducing their TER and increasing epithelial barrier permeability.

### CMV-induced proinflammatory cytokines contribute to the disruption of the intestinal epithelial barrier

**Fig 8:**
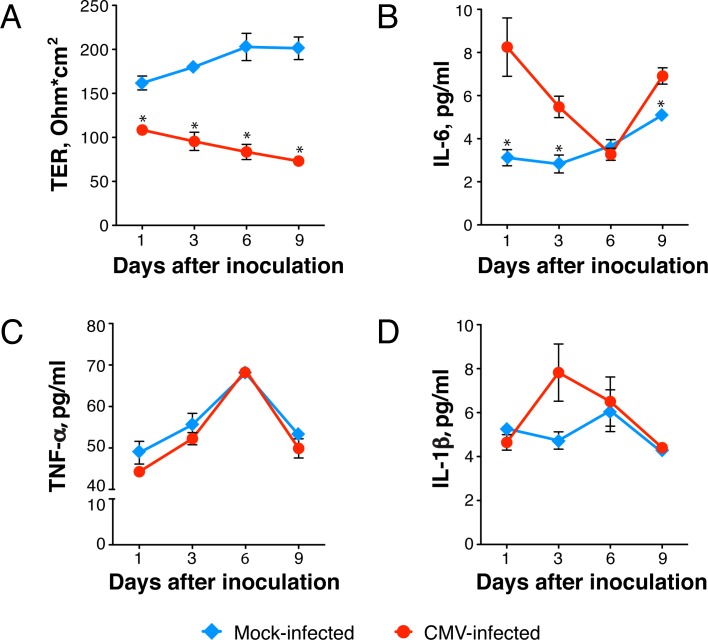
CMV infection modulates proinflammatory cytokine expression. Polarized HCoEpiC were inoculated with CMV VR1814 at a MOI of 1.0 or mock infected. TER was measured by Millicell-ERS every 3 days (**A**), and culture medium was collected for measurement of IL-6 (**B**), TNF-*α* (**C**), and IL-1β (**D**) by ELISA. Data are representative of at least two independent experiments using HCoEpiC derived from two donors and are shown as the mean ± SEM with n = 4 for each experimental condition. Statistical comparison was performed by the Mann Whitney U-test and was considered significant at *P<0.05 (**A**, **B**).

CMV is an inducer of TNF*-α* and IL-1β in the monocyte cell line THP-1 [59, 60] and of IL-6 in peripheral blood mononuclear cells, endothelial cells, and lung fibroblasts [61–63]. On the other hand, it is well known that epithelial tight junction integrity and barrier permeability could be compromised by proinflammatory cytokines TNF*-α*, IL-6, and IL-1β [64–66]. Therefore, we investigated whether CMV infection induced expression of these proinflammatory cytokines in intestinal epithelial cells. Polarized HCoEpiC were mock or CMV infected; TER was monitored every 3 days; and IL-6, TNF-*α*, and IL-1β in the culture medium were measured by ELISA (Fig 8). Over 9 days after inoculation, we observed a gradual decline of TER that was significantly lower compared with mock-infected cells (P<0.05) and indicated CMV-induced impairment of polarized monolayer barrier function (Fig 8A). In parallel, we observed increased production of IL-6, but not TNF-*α* or IL-1β (Fig 8B–8D). IL-6 was detected 1 day after inoculation, and peak levels were 2.7-fold higher than in mock-infected controls (P<0.05). A significant increase in IL-6 relative to mock-infected controls was also observed at days 3 and 9 (P<0.05) and although IL-1β showed a trend toward an increase at day 3, the differences were not statistically significant.

**Fig 9:**
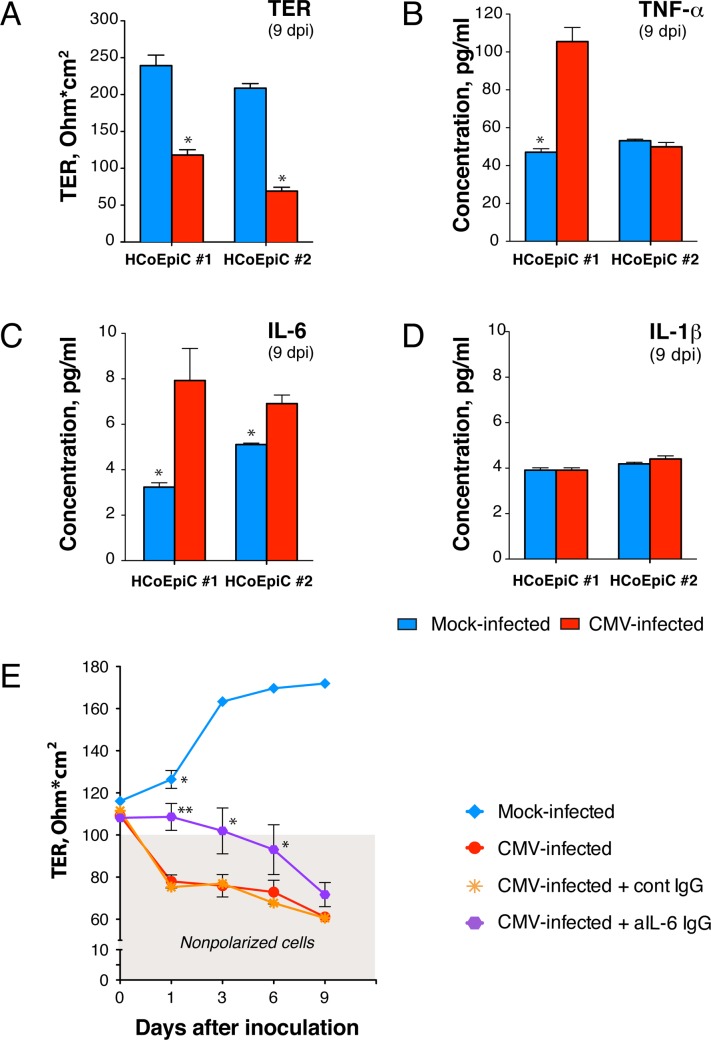
CMV-associated disruption of polarized HCoEpiC monolayer integrity is partially attributed to CMV-induced IL-6. Polarized HCoEpiC derived from two donors (#1 and #2) were inoculated with CMV VR1814 at a MOI of 1.0; TER was measured 9 days after inoculation by Millicell-ERS (**A**), and culture medium was collected for quantification of TNF-*α* (Β**),** IL-6 (**C**), and IL-1β (**D**) by ELISA. (**E**) Polarized HCoEpiC were inoculated with CMV at a MOI of 5.0 or mock infected. Some infected cells were treated before and during infection with anti-human IL-6 mouse IgG or isotype control IgG at 0.1 mg/ml (5 times the 50% neutralization dose). The shaded area (gray) indicates TER of nonpolarized cells. Data are shown as the mean ± SEM with n = 4 (**A**-**D**) and n = 6 (**E**) for each experimental condition. Statistical comparison was performed by the Mann Whitney U-test and was considered significant at (**A**-**D**) *P<0.05; (**E**) **P<0.01 (CMV infected and treated with anti-IL-6 IgG versus CMV infected and treated with isotype control IgG at day 1), *P<0.05 (CMV infected and treated with anti-IL-6 IgG versus CMV infected and treated with isotype control IgG at day 3 and 6; mock infected versus CMV infected and treated with anti-IL-6 IgG at day 1). Dpi, days postinfection.

To assess donor-to-donor variation in proinflammatory cytokine production, we further compared HCoEpiC derived from two donors (Fig 9A–9D). As shown for day 9 after inoculation in Fig 9A, TER was decreased by 2.7-fold in cells derived from donor 1 and 3-fold in cells from donor 2 (P<0.05 compared to mock infected). In cells derived from donor 1, CMV significantly elevated TNF-*α* by 2.1-fold (P<0.05) and IL-6 by 2.5-fold (P<0.05) but did not elevate IL-1β (Fig 9B–9D). In cells derived from donor 2, CMV significantly induced IL-6 by 1.4-fold (P<0.05), but not TNF-*α* nor IL-1β.

Since IL-6 was induced by CMV in cells derived from both donors, we further investigated its role in function-inhibiting experiments (Fig 9E). To determine whether IL-6 is required for CMV-induced disruption of the intestinal epithelial barrier, we used anti-IL-6 neutralizing antibody to block IL-6 function. To stimulate a higher level of IL-6 production, polarized HCoEpiC cells were inoculated with a higher dose of virus at a MOI of 5.0 and were treated before and during CMV infection with mouse IgG anti-human IL-6 or isotype control IgG. Although anti-IL-6 neutralizing antibody were able to prevent, at least partially, CMV-induced disruption of polarized HCoEpiC integrity over 6 days after inoculation (P<0.05), the most significant effect was observed the day after inoculation when TER of the treated HCoEpiC monolayers was 1.4-fold higher than in cells treated with isotype control IgG (P<0.01). On the other hand, the TER of CMV-infected cells treated with anti-IL-6 IgG at this time point was still significantly lower than TER of mock-infected cells (P<0.05). These results indicate that CMV-associated disruption of colonic epithelium integrity could be attributed only to some extent to CMV-induced IL-6.

Taken together, these data indicate that infection of polarized colon epithelial cells with CMV VR1814 induces rapid (within 24 h) release of IL-6, which is able to significantly reduce the TER of the monolayer, thus diminishing epithelial barrier function. Importantly, the novel drug letermovir itself had no effect on the integrity of polarized colon epithelial cells and was able to largely protect colonic epithelial barriers from CMV-induced dysfunction.

## Discussion

**Fig 10:**
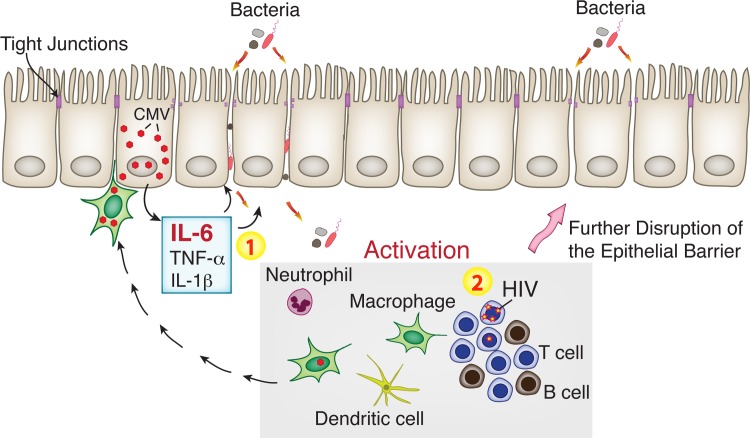
CMV reactivation in the gut initiates bacterial translocation locally, leading to persistent inflammation. Intestinal macrophages with reactivated CMV spread virus to susceptible intestinal epithelial cells. CMV-infected intestinal cells release proinflammatory cytokines, such as IL-6 and possibly TNF-*α* and IL-1β, leading to disruption of tight junctions (1). This results in increased permeability of the intestinal barrier for bacteria and microbial products followed by infiltration of immune cells. Additionally, proinflammatory cytokines released from CMV-infected cells may activate latent HIV provirus and enhance HIV replication (2). Activated immune cells accelerate further disruption of the intestinal epithelial barrier leading to persistent inflammation in the gut of ART-suppressed individuals.

During HIV infection, immune activation linked to intestinal epithelial barrier dysfunction persists despite potent suppressive ART [5, 6, 10, 67–69]. The underlying mechanisms are complex and remain unclear, but the role of opportunistic viral pathogens in the gut has yet to be fully appreciated. Here we report that CMV persists in the rectosigmoid tissues of asymptomatic CMV-positive individuals with both untreated and ART-suppressed HIV infection and that CMV infection coexists with discontinuous epithelial tight junctions. Independent of HIV, CMV impairs the integrity of polarized human intestinal cells, significantly reducing transepithelial electrical resistance and enhancing epithelial barrier permeability. CMV-associated disruption of intestinal epithelium integrity was mediated, at least in part, by CMV-induced IL-6. These observations suggest that CMV reactivation in the gastrointestinal epithelium of HIV-infected individuals could be a potent cofactor that stimulates release of proinflammatory cytokines from intestinal epithelial cells, compromising barrier function and locally initiating bacterial translocation that leads to chronic inflammation in the gut (Fig 10).

Cytomegalovirus, as an opportunistic pathogen in HIV-infected individuals [70], was the cause of significant morbidity and mortality before the introduction of ART [26, 27]. In the era of effective ART, CMV remains an important cofactor in HIV disease progression [30, 71–74] [37, 38], showing strong association with systemic inflammation [28, 29], aging physiology [29, 31, 32], and cardiovascular disease [20, 33–35]. Importantly, in ART-suppressed individuals, T-cell reconstitution in the gastrointestinal tract never reaches the levels observed in uninfected healthy persons [22–25] and could thus be an important site of CMV reactivation [75]. Indeed, the persistent T-cell activation in HIV-infected individuals on ART that is associated with decreased gains in CD4^+^ T-cell counts [76] and mortality in some, but not all, studies [4, 77, 78] was reduced by valganciclovir, a potent anti-CMV nucleoside analog [28]. In our study, we detected numerous HIV RNA-positive T cells and macrophages in the GALT (Fig 1A–1C) of untreated HIV/CMV-coinfected participants, suggesting ongoing elimination of CD4^+^ cells that allows CMV reactivation. In rectosigmoid biopsies from ART-suppressed HIV/CMV-coinfected individuals, we also observed occasional HIV RNA-positive T cells and macrophages, indicating continued low levels of HIV expression in the gut that may promote continued T-cell depletion (Fig 2F).

Active CMV infection (determined by CMV antigenemia and a tissue biopsy specimen that is positive by either culture or IHC staining) is a well-recognized cause of symptomatic colitis in untreated HIV-infected individuals with advanced AIDS. We show here that CMV also persists in the rectosigmoid tissues of asymptomatic CMV-positive participants with both untreated and ART-suppressed HIV infection (Table 1). In untreated HIV infection, CMV proteins were detected in intestinal epithelial cells and in leukocytes located within the mucosal epithelium (Fig 1D–1G). In ART-suppressed HIV infection, both CMV proteins and viral DNA were detected in the intestinal epithelial cells in 9 of 19 (47%) biopsies, suggesting that the intestinal epithelium supports CMV replication (Fig 2A–2E). Importantly, CMV caused a patched pattern of infected cells, a finding that suggests that the lack of CMV proteins or DNA in the other 10 biopsies may represent a sampling error, especially because such small pieces of tissue are taken from one of the largest organs in the body. Thus, we cannot exclude the possibility that CMV was indeed present in the gut of the ART-suppressed HIV/CMV-coinfected individuals who had CMV-negative biopsies in our experiments. The small pinch biopsies may not be fully representative of all gut areas, and CMV shedding may be intermittent, limiting our tissue analyses. On the other hand, the detection of even a few infected cells in these limited areas could reflect the presence of a very large number of CMV-infected cells throughout the intestine.

Our finding that CMV infection in the gut biopsies of asymptomatic CMV-positive participants with both untreated and ART-suppressed HIV infection coexists with discontinuous epithelial tight junctions was especially intriguing. Tight junctions are a distinguishing structural feature of polarized epithelial cells and are crucial for the formation their barrier function [47]. We found here that the typically very distinct apical localization of the junctional adaptor protein ZO-1 in epithelial cells of the intestinal crypts was impaired in the regions where CMV proteins were detected (Fig 3). Importantly, when CMV replication was suppressed with valganciclovir, all intestinal crypts had well-developed junctions with ZO-1 immunolabeling strictly limited to the tight junction regions (Fig 3F). These observations were notably similar to our data for CMV infection in polarized HCoEpiC (Fig 5E and Fig 7).

HIV has well-established cofactor relationships with CMV [70]. Asymptomatic shedding of CMV in the male and female genital tract of ART-suppressed HIV-infected individuals is associated with increased systemic immune activation and higher levels of HIV DNA in peripheral CD4^+^ cells [56, 72, 79]. In accordance with that observation, we noticed a trend toward association between the presence of CMV DNA/proteins and HIV RNA in the rectosigmoid tissues of asymptomatic CMV-positive participants with ART-suppressed HIV infection (Table 1). Although the differences did not reach statistical significance in this small set of participants, we anticipate that future study with larger numbers of participants will better define the link between CMV and HIV replication in the gut. Nevertheless, our finding that ZO-1 staining was intact in the crypts of rectosigmoid biopsies from 4 HIV-negative CMV-positive individuals (Fig 3D) supports this link. Despite detection of numerous CD68/CD163-positive macrophages containing CMV proteins in these biopsies, no spread of CMV infection to intestinal epithelial cells was detected. These observations suggest that HIV indeed is associated with an increase of CMV infection concurrent with damage to the intestinal epithelium.

To define the role of intestinal epithelial cells during gastrointestinal CMV disease independent of HIV, we further investigated their susceptibility to CMV infection using a SCID-hu gut mouse model reported previously [80–83]. We observed extensive damage of the mucosal epithelium 7 days after inoculation with CMV (Fig 4C), and infection was indicated by the abundant expression of CMV IE proteins. The lumen of damaged intestinal crypts contained numerous IE-positive cytomegalic cells (Fig 4D), mirroring the shedding of cytomegalic cells observed in the gut biopsies with clinically diagnosed CMV colitis (Fig 3E). We are intrigued by the high level of susceptibility of the intestinal epithelium to CMV *in vivo* and our finding that CMV persists in the intestinal epithelium of HIV/CMV-coinfected individuals. To focus further on the study of CMV pathogenesis exclusively in intestinal epithelial cells, we modeled CMV infection *in vitro* by the use of primary colon epithelial cells. Although CMV disease can involve any region of the gastrointestinal tract in AIDS patients, the colon is the site most frequently infected [40–42, 84]. Much of our knowledge about CMV pathogenesis in the intestinal epithelium comes from studies conducted in the Caco-2 cell line of heterogeneous human epithelial colorectal adenocarcinoma cells [85, 86]. The permissiveness of Caco-2 cells to various laboratory strains of CMV is controversial, so we performed our study in primary colon epithelial cells (HCoEpiC) using VR1814, a clinical isolate of CMV that was adapted for growth in human umbilical vein endothelial cells (HUVEC) [87] and maintained at low passages 27–29. These cells formed polarized monolayers when plated on collagen-coated transwell inserts for 6 days, expressing ZO-1 in a ring-like pattern (Fig 5D), and they were permissive to CMV replication producing infectious viral progeny (Fig 5E–5H). Experiments with CMV Towne in polarized Caco-2 cells inoculated at a MOI of 25 showed that this strain preferentially infects from the basolateral surface. In contrast, we found that the CMV strain VR1814 impaired integrity of polarized HCoEpiC monolayers to a greater extent when the cells were inoculated at a MOI of 1.0 from the apical surface (Fig 6). Basolateral entry of CMV into polarized HCoEpiC recapitulates hematogenous virus spread because the basolateral surface of the mucosal epithelium is oriented toward the underlying blood vessels of the lamina propria. Apical entry of CMV into polarized HCoEpiC may resemble both homosexual virus transmission via semen [88–91] and postnatal CMV transmission via breast milk in premature infants [92–94]. The latter is very intriguing because HCoEpiC cells were derived from the fetal intestine, therefore CMV entry through the apical surface could be specific to fetal/neonatal cells.

We and others have previously reported that CMV disrupts the tight junctions of polarized epithelial cells and the adherens junctions of polarized endothelial cells [48–52]. In Caco-2 cells, CMV disrupted polarized monolayers and decreased TER beginning 12 days after inoculation [85]. In our experiments, CMV disrupted tight junctions of polarized HCoEpiC and significantly reduced TER by 1 day after inoculation, demonstrating the high degree of virulence of VR1814 in primary colon epithelial cells (Fig 7A).

Notably, the novel anti-CMV drug letermovir, which exhibits potent antiviral activity against clinical isolates [58, 95], prevented damage of the HCoEpiC monolayer to a level that was sufficient to maintain cell polarity (Fig 7A and 7C) and to inhibit CMV-induced “leakage” of FITC-dextran (Fig 7D). Importantly, in contrast to GCV, letermovir itself had no effect on the integrity of mock-infected polarized HCoEpiC (Fig 7B). Of note, both drugs were not able to prevent the initial small TER declines at day 1 after CMV inoculation (Fig 7A). GCV targeting viral polymerase UL54 acts at early steps of the CMV replication cycle [96]. Letermovir is known to act at a late stage of CMV replication that involves viral DNA processing and/or packaging without inhibiting *de novo* synthesis of CMV DNA and protein expression [95]. We suggested earlier that increased barrier permeability of polarized retinal pigment epithelial cells was mediated by the CMV protein US9 [49, 97]. Since CMV US9 gene expression peaks 24 h after infection [98], it would be interesting to determine whether US9 or other CMV early genes could be directly involved in the disruption of intercellular tight junctions of the intestinal epithelium.

CMV is a well-known inducer of TNF*-α* and IL-1β in the monocyte cell line THP-1 and of IL-6 in peripheral blood mononuclear cells, lung fibroblasts, and endothelial cells [59–63]. Moreover, TNF-*α* transcripts are abundantly expressed in colonic mucosa from untreated AIDS patients with CMV colitis and are associated with macrophage-like cells containing cytomegalic inclusions [99]. Importantly, upregulation of those proinflammatory cytokines has been attributed to CMV IE gene expression, and these cytokines increase paracellular permeability of epithelial and endothelial cells [57, 100–109]. It is interesting that despite the induction of proinflammatory cytokines early after infection, CMV suppresses TNF*-α* and IL-1β signaling pathways at late times, demonstrating unique adaptation capacities that enable virus persistence within the host [110]. Altogether, these published observations suggest that CMV-induced disruption of intestinal tight junctions could be mediated by these proinflammatory cytokine pathways. Indeed, in our experiments we found that the initial drop in HCoEpiC TER at 24 h after CMV inoculation was accompanied by a significant increase (P<0.05) in secretion of IL-6 (Fig 8A and 8B). The role of CMV-induced IL-6 in this process was confirmed by experiments demonstrating an increase in TER when cells were treated before and during infection with IL-6-neutralizing antibodies (Fig 9E). At 24 h, the TER of mock-infected cells was still higher than CMV-infected cells in the presence of anti-IL-6 antibodies (P<0.05), suggesting that other CMV-induced factors or viral genes could be involved in the modulation of intestinal epithelium permeability. We anticipate that future studies will better define those additional factors. Nevertheless, our results showed that CMV-associated disruption of colon epithelial integrity could be attributed, at least in part, to CMV-induced IL-6. Notably, HIV infection does not induce IL-6 expression *in vitro* [111], although increased plasma IL-6 levels have been associated with HIV disease progression risk [1, 112–116]. The exact cause of elevated plasma IL-6 in chronic HIV disease remains unclear, but it could result from a combination of factors. Our data indicate that persistent CMV replication in various sites of HIV-infected individuals could be one of these factors.

Others have reported that active CMV infection could be often detected in the intestine of individuals with inflammatory bowel diseases and may contribute to the inflammatory process through virus-induced IL-6 [55]. It is interesting to note that intestinal tissue samples in that prior study included 10 gut biopsies from CMV-infected AIDS participants, and CMV-specific antigens were detected in all 10 samples, 4 of which were double-positive for both CMV and IL-6. Furthermore, CMV shedding was associated with higher IL-6 levels in vaginal swabs after initiation of ART in HIV-infected women [56]. We also found a trend toward higher IL-6 plasma level in samples obtained from participants with CMV-positive gut biopsies compared to CMV-negative biopsies, but the differences did not reach statistical significance. These results may reflect the fluctuating levels of IL-6 that we observed in our *in vitro* HCoEpiC time-course experiments (Fig 8B) and indicate variable levels of IL-6 in various tissues containing CMV foci. Nevertheless, the modest changes in IL-6 plasma levels in this small set of participants are highly intriguing, and we anticipate that future study with larger numbers of participants will better define the relationship between plasma IL-6 and the presence of CMV in the gut.

It is equally interesting to consider the role of CMV-induced IL-6 in increased epithelial proliferation in ART-treated and untreated individuals with intestinal epithelial barrier dysfunction [6]. We reported earlier that disruption of adherens junctions of polarized endothelial cells by CMV triggered proliferation of bystander endothelial cells [51], suggesting a balance between lysis of infected endothelial cells and replacement by uninfected ones [117]. It has also been reported that the CMV-encoded chemokine receptor US28 mediates cell proliferation through activation of the IL-6-STAT3 signaling axis [118] and that the CMV secretome promotes angiogenesis and lymphangiogenesis through IL-6 signaling [119, 120]. Based on these published reports, we speculate that CMV infection of the intestinal epithelium may trigger IL-6 production, resulting in enhanced cell proliferation to replace infected intestinal cells. Experiments with polarized HCoEpiC revealed cytomegalic gB-expressing cells positioned above the cell monolayer (Fig 5H), mimicking the shedding of cytomegalic cells in the gut of an individual with CMV colitis (Fig 3E) and in the SCID-hu gut infected *in vivo* with CMV (Fig 4D). Furthermore, CMV slowly replicated in polarized HCoEpiC releasing low levels of viral progeny (Fig 5F). These findings suggest that CMV does not lyse all the cells in foci of polarized intestinal cells. Detached infected cells could be replaced by more permissive nonpolarized proliferating cells (Fig 5C), creating small foci of CMV infection with compromised tight junctions that may locally facilitate bacterial translocation leading to persistent inflammation [10].

AIDS-associated gastrointestinal CMV disease typically responds well to ganciclovir and foscarnet [121, 122]. However, the impact of CMV on the junctions of the intestinal epithelium in the presence of currently approved or novel HCMV drugs has never before been studied. Here we found that CMV-induced disruption of polarized HCoEpiC monolayer integrity could be prevented by the novel small-molecule CMV terminase inhibitor letermovir [58] (Fig 7A and 7C). Despite the observation that the TER of CMV-infected cells in the presence of letermovir was still below the level of mock-infected cells, it exceeded the 100 Ohm*cm^2^ threshold, indicating that cells maintained their intact junctions and cell polarity. Letermovir is a new, anti-CMV agent currently being developed for the prevention of CMV disease in immunocompromised transplant patients [123, 124]. Importantly, letermovir has no antagonism across a broad panel of commonly used anti-HIV drugs and was suggested for treatment of active CMV infection in HIV/CMV-coinfected individuals [125]. Our finding that attenuation of CMV infection by letermovir was sufficient to prevent significant CMV-induced declines in TER supports development of novel interventional strategies to increase intestinal epithelial barrier function in HIV infection.

In summary, in this paper we addressed the role of an important opportunistic pathogen, CMV, as a cofactor of intestinal barrier dysfunction in asymptomatic HIV infection by several approaches. First, we used a combination of state-of-the-art ISH technology (RNAscope) and immunohistochemistry to demonstrate that CMV persists in the gut of CMV-positive participants with both untreated and ART-suppressed HIV infection and is associated with the disruption of epithelial tight junctions. We then used two different model systems: polarized primary human intestinal cells and a mouse model of human gut to assess the impact of CMV on the intestinal epithelium independently of HIV. We found that CMV impairs the integrity of intestinal epithelial cells, reducing transepithelial electrical resistance and enhancing epithelial barrier permeability, and that this effect is mediated, at least in part, by CMV-induced IL-6. These results support our hypothesis that CMV reactivation in the gastrointestinal epithelium of HIV-infected individuals is a potent cofactor that stimulates release of proinflammatory cytokines from intestinal epithelial cells, compromising their barrier function, and initiating localized bacterial translocation leading to chronic inflammation in the gut. Moreover, we showed that CMV-associated disruption of intestinal epithelium integrity could be largely prevented by letermovir, a novel anti-CMV drug currently in clinical development. Thus, the addition of letermovir to a suppressive ART regimen might be explored as a strategy to reduce microbial translocation and systemic immune activation in future trials. Altogether, our results provide further evidence that CMV remains an important cofactor in HIV pathogenesis even during ART-mediated HIV suppression and suggest new antiviral interventions to prevent gut epithelial barrier dysfunction in treated HIV infection.

## Materials and methods

### Ethics statement

Archived rectosigmoid biopsy samples were from the SCOPE cohort at the University of California, San Francisco (UCSF). The SCOPE cohort is an ongoing longitudinal study of over 1,500 HIV-infected and uninfected adults followed for research purposes. The UCSF Committee on Human Research reviewed and approved the SCOPE study (IRB# 10–01218), and all participants provided written informed consent.

Protocols involving animals were approved by the UCSF Institutional Animal Care and Use Committee (#AN111327-02). This protocol adheres to the National Institutes of Health’s *Public Health Service Policy on Humane Care and Use of Laboratory Animals*.

### Gut biopsy sample collection

Archived rectosigmoid biopsy samples were retrieved from the SCOPE cohort at UCSF as previously described [6]. For SCOPE studies, participants were recruited based on CD4 count, duration of ART, and HIV infection and treatment status, and they had no symptoms or suspicion of CMV disease.

Using this cohort, archived gut biopsies and plasma samples were selected from 5 HIV-negative, CMV-negative controls; 3 CMV-positive, HIV-viremic untreated individuals; 19 CMV-positive, ART-suppressed individuals; and 4 HIV-negative, CMV-positive individuals (Table 1). Two samples were from an individual with clinically diagnosed active CMV infection before and after valganciclovir treatment. Prior to sigmoidoscopy and gastrointestinal biopsy, study participants underwent a blood draw and received a Fleet enema. CD4^+^ T-cell counts (cells/mm^3^) and CMV serology (CMV IgG) were performed by a Clinical Laboratory Improvement Amendments (CLIA)-certified clinical laboratory located at Zuckerberg San Francisco General. Rectosigmoid biopsies (~3 mm in diameter) were obtained 10–20 cm from the anus using jumbo forceps, and four biopsies were formalin fixed and paraffin embedded for IHC and ISH.

### SCID-hu gut mice

Fetal gut tissues (18–24 g. w.) were obtained from women with normal pregnancies before elective termination for nonmedical reasons with informed consent according to local, state, and federal regulations. Single intact segments of the human fetal intestine (2−3 cm in length) were transplanted subcutaneously on the back of 6–8-week-old male C.B17 scid mice (C.B-*Igh-1*^*b*/IcrTac^-*Prkdc*^*scid*^ Taconic) [83]. Mice were maintained under specific pathogen-free conditions. Four weeks after gut implantation, mice were anesthetized, and implants were inoculated with 5.7–6.4 log_10_ IU of CMV by direct injection into the gut lumen. At 7 and 14 days after inoculation, mice were euthanized, and implants were dissected and fixed in 3.7% formaldehyde solution (Sigma-Aldrich) for immunohistochemistry.

### Virus and cells

VR1814, a clinical isolate of CMV that was adapted for growth in HUVEC [87], was a generous gift (at passage 23) from Dr. Maria Grazia Revello. Virus was propagated in HUVEC (Lonza), and viral stocks at passages 27–29 were prepared from supernatant virus [126, 127]. The titers of infectious virus were determined by a rapid method of immunological detection and quantification of CMV IE proteins that has been shown to correlate with the conventional plaque assay [126, 128]. Neonatal human dermal fibroblasts (NHDF-Neo) (Lonza) were used as the indicator monolayer for the assay, and virus titers are expressed as IU [126]. HCoEpiC isolated from human fetal colonic tissue were purchased from ScienCell at passage 1. HCoEpiC were cultured in colonic epithelial cell medium (CoEpiCM, ScienCell), and all experiments were performed at passage 2 or 3. The purity of colonic epithelial cells was verified at each passage by immunofluorescence staining of formaldehyde-fixed cytospin preparations with a rabbit monoclonal antibody to human cytokeratin 19 (Abcam) (S3 Fig).

### Generation of a polarized monolayer of the colonic epithelial cells

To form polarized monolayers, HCoEpiC at passage 2 were seeded at 3 x 10^4^ cells/cm^2^ on either the upper or lower surface of 6-mm-diameter transwell permeable membranes (0.45 μm pore size, Costar) coated with 30 μg/ml rat-tail type 1 collagen (BD Biosciences) for 30 min at 37°C. To seed the cells on the lower surface, the transwell insert was inverted for 2 h to allow the cells to attach. The monolayers were grown for at least 6 more days with fresh medium added every 2 days.

### Assay for transcellular electrical resistance

TER of cells grown on transwell permeable membranes was measured with a Millicell ERS-2 voltohmmeter (Millipore). The value obtained from a blank insert coated with collagen was subtracted to obtain net resistance, which was multiplied by the membrane area to yield the resistance in area-corrected units. TER values exceeding the 100 Ohm*cm^2^ threshold indicated that cells had developed tight junctions and were polarized. The polarity of HCoEpiC monolayers was verified in parallel by fluorescence immunolocalization of ZO-1, a marker of tight junctions (Fig 5D). The functional integrity of the tight epithelial monolayer was further confirmed by the lack of FITC-dextran leakage in the transepithelial permeability assay.

### Transepithelial permeability assay

The permeability of the polarized monolayers was quantified by measuring the transepithelial flux of 4-kDa FITC labelled dextran (Sigma-Aldrich). Briefly, following polarization, 150 μl of Hank’s balanced salt solution (HBSS) containing FITC-dextran (1 mg/ml) was applied to the upper chamber of the transwell, and 450 μl of HBSS was added to the lower chamber. As a positive control (permeable monolayer), 1% Triton X-100 (Sigma-Aldrich) in HBSS was used to destroy epithelial integrity. Following incubation at 37°C for 2 h, 100 μl of the HBSS from the lower chamber was transferred into a translucent 96-well plate to measure relative fluorescence units (RFU) in triplicate. A fluorescence standard curve was prepared with fixed concentrations of FITC-dextran in HBSS from 200–3.125 μg/ml for subsequent extrapolation of unknown FITC-dextran concentrations recovered from the basolateral transwell compartment. RFU was measured with a SpectraMax M2 multimode microplate reader (Molecular Devices) with excitation wavelength of 490 nm and emission wavelength of 520 nm.

### Cell treatments

To suppress CMV replication after virus adsorption, polarized HCoEpiC were treated with inhibitory concentrations (10 times the IC_50_) of letermovir (50 nM) (MedchemExpress, LLC) or GCV (20 μM) (Invivogen, Inc.) as described [58]. Since letermovir was dissolved in DMSO, a vehicle control (0.001% DMSO) was included. The effect of IL-6 was studied in function-inhibiting experiments using a neutralizing anti-human IL-6 mouse monoclonal antibody (Abcam). Before and during infection, polarized HCoEpiC were incubated with anti-human IL-6 at 0.1 mg/ml (5 times the 50% neutralization dose) as described [129]. Low-endotoxin, azide-free isotype control mouse IgG (Abcam) at the same concentration was used as a control.

### Immunohistochemistry

NHDF-Neo used as the indicator monolayer for titration of infectious CMV, and HCoEpiC cytospins were fixed with ice-cold 70% methanol (Sigma-Aldrich) for 20 min. Polarized HCoEpiC in transwells were fixed in 3.2% paraformaldehyde (Electron Microscopy Sciences) for 15 min at room temperature and then permeabilized for 5 min in 0.1% Triton-X-100 (Sigma-Aldrich). Gut implants were fixed in 3.7% formaldehyde (Sigma-Aldrich) and infiltrated with 5–15% sucrose followed by embedding in optimal-cutting-temperature compound and freezing in liquid nitrogen [130]. Tissue sections of archived FFPE biopsy samples were deparaffinized in xylene and rehydrated in graded alcohols. Endogenous peroxidase was quenched with 3% H_2_O_2_ in methanol for 10 min, and heat-induced epitope-retrieval using 10 mM sodium citrate buffer (pH 6.0) was performed in a DC2002 decloaking chamber (Biocare Medical). For ZO-1 detection, antigen retrieval was followed by permeabilization with 0.2% Triton X-100 for 45 min. Primary antibodies included a mouse monoclonal to CMV IE and a blend (Millipore) of anti-CMV monoclonal antibody clone 8B1.2 reacting with an IE protein of 68–72 kDa, clone 1G5.2 reacting with an unspecified late CMV protein, and clone 2D4.2 reacting with a late structural protein of 47–55 kDa; a rabbit monoclonal specific for human cytokeratin 19 (ab76539) from Abcam; and rabbit polyclonals to ZO-1 from Invitrogen. Primary antibody binding to its target antigen was detected by chromogenic or fluorescent methods. For chromogenic detection, secondary antibodies were labeled with horseradish peroxidase (HRP) or alkaline phosphatase (AP) using ImmPRESS reagents (Vector Laboratories). Chromogenic ImmPACT DAB and SG HRP substrates and ImmPACT Red AP substrate were also from Vector Laboratories. Counterstaining for chromogenic detection was performed with hematoxylin (Vector Laboratories). For fluorescence detection, secondary antibodies labeled with Alexa Fluor 488 or Alexa Fluor 594 were obtained from Jackson ImmunoResearch, and diamidino-2-phenylindole (DAPI) counterstain was obtained from Vector Laboratories. For double immunolabeling, cells were simultaneously incubated with primary antibodies from various species, with secondary antibodies labeled with HRP or AP for chromogenic detection, and Alexa Fluor 488 or Alexa Fluor 594 for fluorescent detection. The specificity of each immunohistochemical reaction was verified with nonimmune rabbit or mouse IgG (Vector Laboratories) as the primary antibody.

### In situ hybridization

A new generation state-of-the-art ISH technology (RNAscope) developed by Advanced Cell Diagnostics employs a unique “double Z” probe design that greatly increases signal-to-noise ratio enabling the visualization of single transcripts. RNAscope was performed using a 2.0HD Reagent Kit-RED kit (Advanced Cell Diagnostics) according to the manufacturer's instructions. HIV-1 RNA was detected using a HIV-1-gagpol probe, which targets gag-pol coding sequence region 587–4601. CMV DNA was detected using HHV5-IE and HHV5-pp65 probes, which target the noncoding sequence regions of CMV strain Merlin UL123 (172678–173852) and UL83 (120742–122152) respectively, all from Advanced Cell Diagnostics. Human peptidyl-prolyl cis-trans isomerase B (PPIB), encoded by the PPIB gene, was detected with the Hs-PPIB probe in HeLa cell line control slides (Advanced Cell Diagnostics) and served as an RNAscope positive control. The RNAscope assay was followed by standard IHC with chromogenic detection.

### Microscopy and image analysis

Tissue analyses were performed blinded to the clinical information and required examination of at least 5 tissue sections. ISH and IHC tissue sections and cells were analyzed with a Leica DM6000 B bright field/fluorescence microscope equipped with a Leica DFC 500 camera. Images were acquired with Leica LAS v4.3 software and analyzed with ImageJ software.

### Measurement of cytokine levels

CMV-infected and mock-infected culture media from polarized HCoEpiC were collected from both the upper and lower chambers of the transwells at various times after inoculation, centrifuged to remove cell debris, aliquoted, and stored at –70°C. Concentrations of TNF*-α*, IL-6, and IL-1β in the culture media and plasma samples were measured by Quantikine ELISA (R&D Systems).

### Statistical analysis

Statistical analysis was performed by GraphPad Prism version 5. All values are expressed as the mean ± standard error of the mean (SEM). Assay results were compared using the nonparametric Mann Whitney U-test and chi-square test, and differences were considered significant if P<0.05.

## Supporting information

S1 Fig HIV detection in control tissues. Thymic organoid tissue from a HIV-infected humanized BLT mouse was used as a positive control (**A**-**E**). RNAscope ISH was followed by colorimetric IHC for human macrophage markers CD68 and CD163. HIV RNA was detected in human CD68^+^CD163^+^ macrophages and CD68^−^CD163^−^ putative T cells (**B**-**E**). (**F**, **G**) HIV RNA was not detected in gut biopsies from CMV-negative, HIV-negative individuals. Nuclei were counterstained with hematoxylin. Scale bars: 200 μm (A, F), 50 μm (G); insets: x630, original magnification.
(TIF)

S2 Fig CMV detection in control tissues. Human fetal lung explant tissue 48 h after ex vivo inoculation with CMV VR1814 at 10^6^ IU/explant was used as a positive control (**A, B,** adjacent sections). DNAscope ISH using probes V-HHV5-IE (**A**) and V-HHV5-pp65 (**B**) targeting CMV IE and pp65 noncoding sequences, respectively, was followed by colorimetric IHC for cytokeratin (CK), an epithelial cell marker. CMV DNA was detected in CK-positive epithelial cells and mostly in CK-negative stromal lung cells. (**C**) CMV DNA was not detected in gut biopsies from HIV-negative CMV-negative individuals. Nuclei were counterstained with hematoxylin. Scale bars: 50 μm.
(TIF)

S3 Fig Verification of the epithelial origin of HCoEpiC. Mock-infected cells at passage 3 (**A**) and inoculated with CMV VR1814 at a MOI of 1.0 (**B**). HCoEpiC expressed cytokeratin, an epithelial cell marker (green) but not vimentin, a mesenchymal cell marker (**C**, green). Nuclei were counterstained with DAPI. Scale bars: 20 μm.
(TIF)
