# Supplementary figures and images for: Replication of CMV in the gut of HIV-infected individuals and epithelial barrier dysfunction
Source: PLoS Pathog. 2017 Feb 27;13(2):e1006202. doi: 10.1371/journal.ppat.1006202 (PMC5328284; doi:10.1371/journal.ppat.1006202)

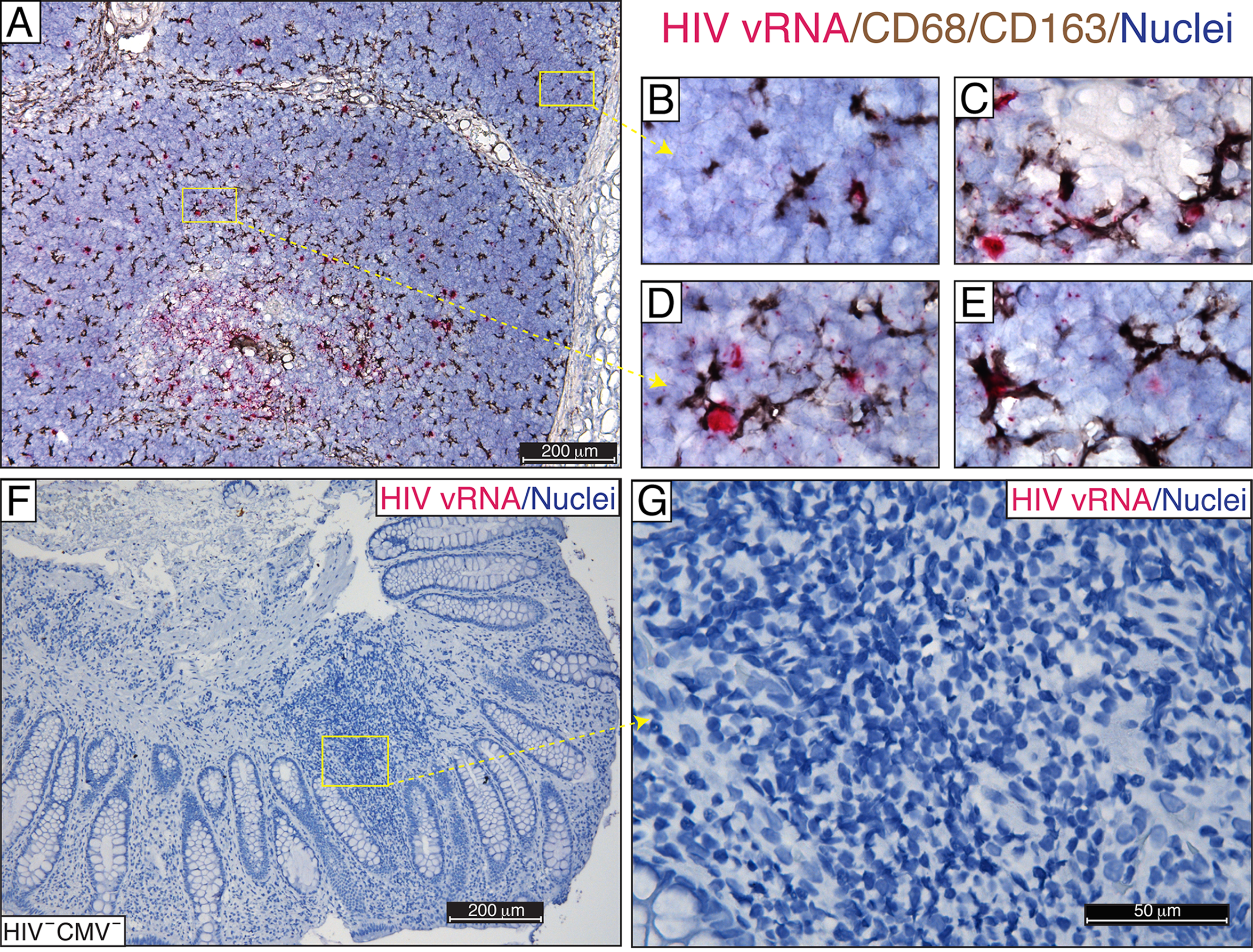

Supplement: S1 Fig — Thymic organoid tissue from a HIV-infected humanized BLT mouse was used as a positive control (A-E). RNAscope ISH was followed by colorimetric IHC for human macrophage markers CD68 and CD163. HIV RNA was detected in human CD68+CD163+ macrophages and CD68−CD163− putative T cells (B-E). (F, G) HIV RNA was not detected in gut biopsies from CMV-negative, HIV-negative individuals. Nuclei were counterstained with hematoxylin. Scale bars: 200 μm (A, F), 50 μm (G); insets: x630, original magnification. (TIF) [file ppat.1006202.s001.tif]

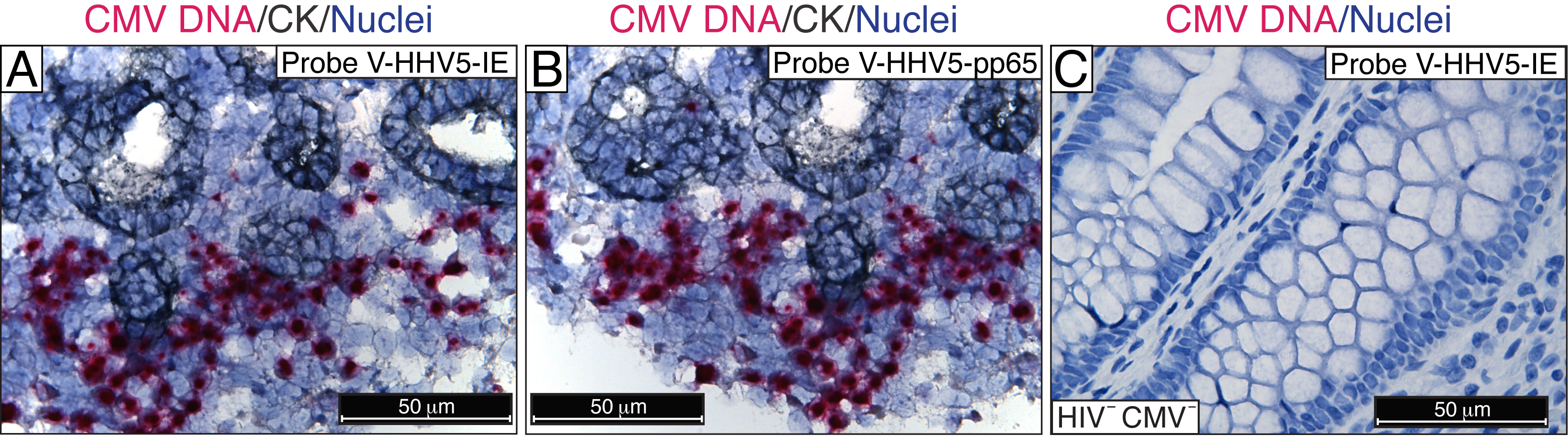

Supplement: S2 Fig — Human fetal lung explant tissue 48 h after ex vivo inoculation with CMV VR1814 at 106 IU/explant was used as a positive control (A, B, adjacent sections). DNAscope ISH using probes V-HHV5-IE (A) and V-HHV5-pp65 (B) targeting CMV IE and pp65 noncoding sequences, respectively, was followed by colorimetric IHC for cytokeratin (CK), an epithelial cell marker. CMV DNA was detected in CK-positive epithelial cells and mostly in CK-negative stromal lung cells. (C) CMV DNA was not detected in gut biopsies from HIV-negative CMV-negative individuals. Nuclei were counterstained with hematoxylin. Scale bars: 50 μm. (TIF) [file ppat.1006202.s002.tif]

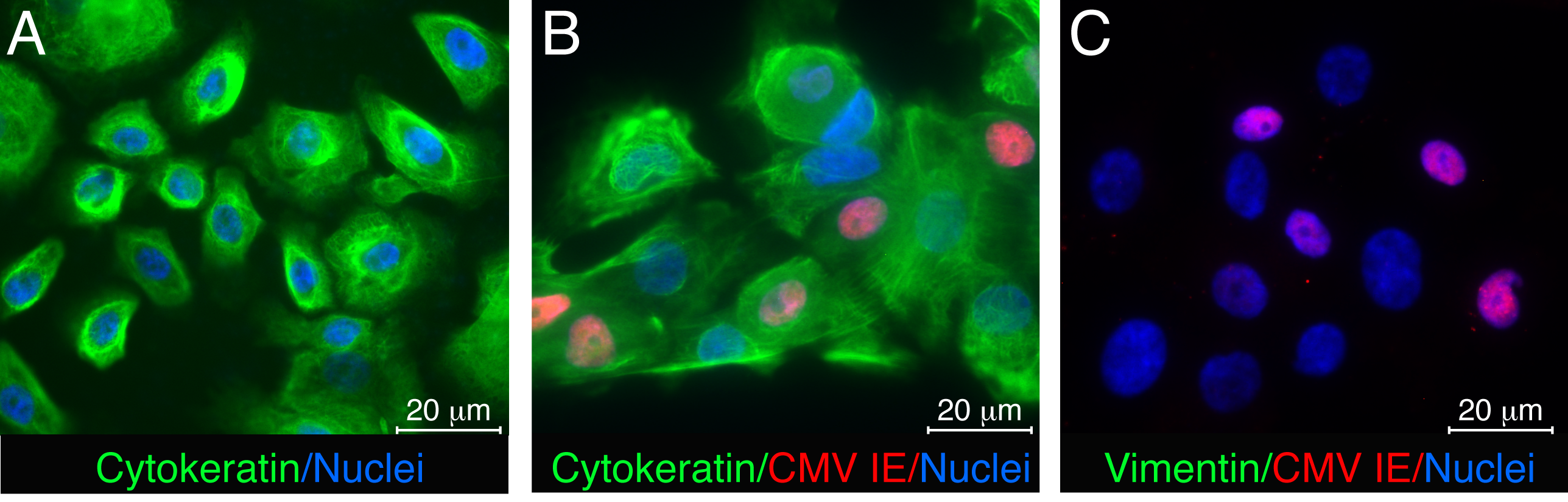

Supplement: S3 Fig — Mock-infected cells at passage 3 (A) and inoculated with CMV VR1814 at a MOI of 1.0 (B). HCoEpiC expressed cytokeratin, an epithelial cell marker (green) but not vimentin, a mesenchymal cell marker (C, green). Nuclei were counterstained with DAPI. Scale bars: 20 μm. (TIF) [file ppat.1006202.s003.tif]
